# Case Report: Ataxia telangiectasia with severe hemorrhagic cystitis

**DOI:** 10.3389/fped.2026.1740485

**Published:** 2026-02-26

**Authors:** Hua Song, Yi Lin, Yuwei Xian

**Affiliations:** 1 Department of Pediatric Surgery, Women and Children’s Hospital, Qingdao University, Qingdao, China; 2 Department of Pediatric Nephrology and Rheumatology, The Affiliated Hospital of Qingdao University, Qingdao, China; 3 Department of Ultrasound, Qingdao Municipal Hospital, Qingdao, China

**Keywords:** ataxia telangiectasia, ATM gene, cystoscopy, hematuria, hemorrhagic cystitis

## Abstract

**Background:**

Ataxia telangiectasia (AT) is a rare autosomal recessive genetic disorder caused by variants in the ataxia-telangiectasia mutated (*ATM*) gene. AT is characterized by progressive cerebellar degeneration, telangiectasia, immunodeficiency, cancer susceptibility, and radiosensitivity. This report presents a case of classic AT complicated by severe hemorrhagic cystitis, a rare clinical manifestation. Genetic analysis revealed novel variants in the *ATM* gene.

**Case presentation:**

A 12-year-old Han Chinese boy presented with recurrent gross hematuria that progressed in frequency and severity after completion of chemotherapy for T-cell acute lymphoblastic leukemia (ALL). He had developed gait instability at age 2, and brain MRI showed cerebellar atrophy. Genetic testing revealed compound heterozygous *ATM* variants: c.8357G>T (p.Gly2786Val) (maternal) and IVS54+3A>C (paternal) (NM_000051). Cystoscopy revealed multiple telangiectatic lesions of the bladder mucosa with associated yellow-brown sedimentation. Emergency cystoscopic electrocoagulation controlled the bleeding.

**Conclusion:**

We report two novel *ATM* variants (c.8357G>T, IVS54+3A>C) in a patient with classic AT who developed severe hemorrhagic cystitis associated with bladder wall telangiectasia. AT patients may be at risk for delayed, potentially life-threatening hemorrhagic cystitis, particularly following cyclophosphamide exposure. Cystoscopy is essential for diagnosis and enables timely endoscopic management.

## Introduction

Ataxia telangiectasia (AT; Louis-Bar syndrome) is a rare autosomal recessive genetic disorder caused by variants in the *ATM* gene, with an estimated prevalence of 1–9/100,000 (1). The *ATM* gene is located at chromosome 11q22-23, comprises 63 exons, and encodes a 3,056-amino-acid serine/threonine kinase belonging to the phosphoinositide 3-kinase (PI3K)-related kinase family. ATM plays a central role in the DNA damage response, particularly in signaling following double-strand DNA breaks. Loss of functional ATM impairs multiple nuclear and cytoplasmic signaling pathways, resulting in multisystem manifestations. The cardinal clinical features include progressive cerebellar ataxia, oculocutaneous telangiectasia, ionizing radiation sensitivity, immunodeficiency, and cancer susceptibility. We present a case of classic AT complicated by recurrent, progressive hematuria due to bladder wall telangiectasia.

## Case report

### Clinical presentation

A 12-year-old Han Chinese boy was referred for recurrent gross hematuria. Initially, episodes occurred every 3–4 months and resolved spontaneously; over time, they became more frequent and eventually occurred approximately every 2 weeks. The hematuria progressed to severe episodes with passage of blood clots.

### Past medical history

The patient exhibited gait instability from age 2 years, with progressive deterioration. Brain MRI demonstrated cerebellar atrophy. At age 8, he presented with a persistent neck mass and was diagnosed with T-cell acute lymphoblastic leukemia (intermediate risk) based on bone marrow morphology, flow cytometry, cytogenetics, and molecular studies.

The patient received chemotherapy for 2.5 years. The regimen included VDLD, CAT [cyclophosphamide (CTX): 1.0 g/m^2^ × 2], HR-1' (CTX: 0.2 g/m^2^ × 4) × 1, HR-2’ [Ifosfamide (IFO): 0.8 g/m^2^ × 5] × 2, HR-3' × 2, VDLD, HD-MTX×2, and VP × 2. Hyperhydration and mesna prophylaxis were administered during CTX/IFO exposure to prevent hemorrhagic cystitis. No hematuria was observed during the administration of CTX or IFO. The latency period between the last administration of CTX or IFO and the onset of hematuria was 1.5 years. Bone marrow remission has been maintained to date.

### Personal and family history

He was the second child in the family, delivered by cesarean section at 28 weeks of gestation. His language and behavioral development were delayed compared to peers; he achieved assisted walking at 17 months. His parents and elder sister are healthy, with no family history of consanguineous marriage or hereditary diseases.

### Physical examination

The patient’s vital signs were as follows: temperature 36.7 °C, pulse 78 bpm, respiration 18/min, blood pressure 87/52 mmHg, and weight 26 kg. The patient exhibited moderate nutritional status. Ocular telangiectasia (dilated capillaries) was observed bilaterally in the sclerae. Cardiac and pulmonary examinations were unremarkable. Neurological examination revealed positive signs for dysmetria and ataxia, including positive finger-to-nose, heel-knee-shin, and rapid alternating movement tests, as well as a positive Romberg sign.

### Investigations and treatment

Complete blood count revealed mild anemia with a normal platelet count; coagulation profiles were normal. Electrolytes, serum chemistries, and liver and kidney function tests were within normal limits. Immunological testing showed a low serum IgG level (4.09 g/L), while IgM and IgA levels were normal. Urinalysis indicated a significant increase in red blood cells, primarily of normal morphology. Urinary ultrasound revealed blood clots within the bladder.

**Figure 1:**
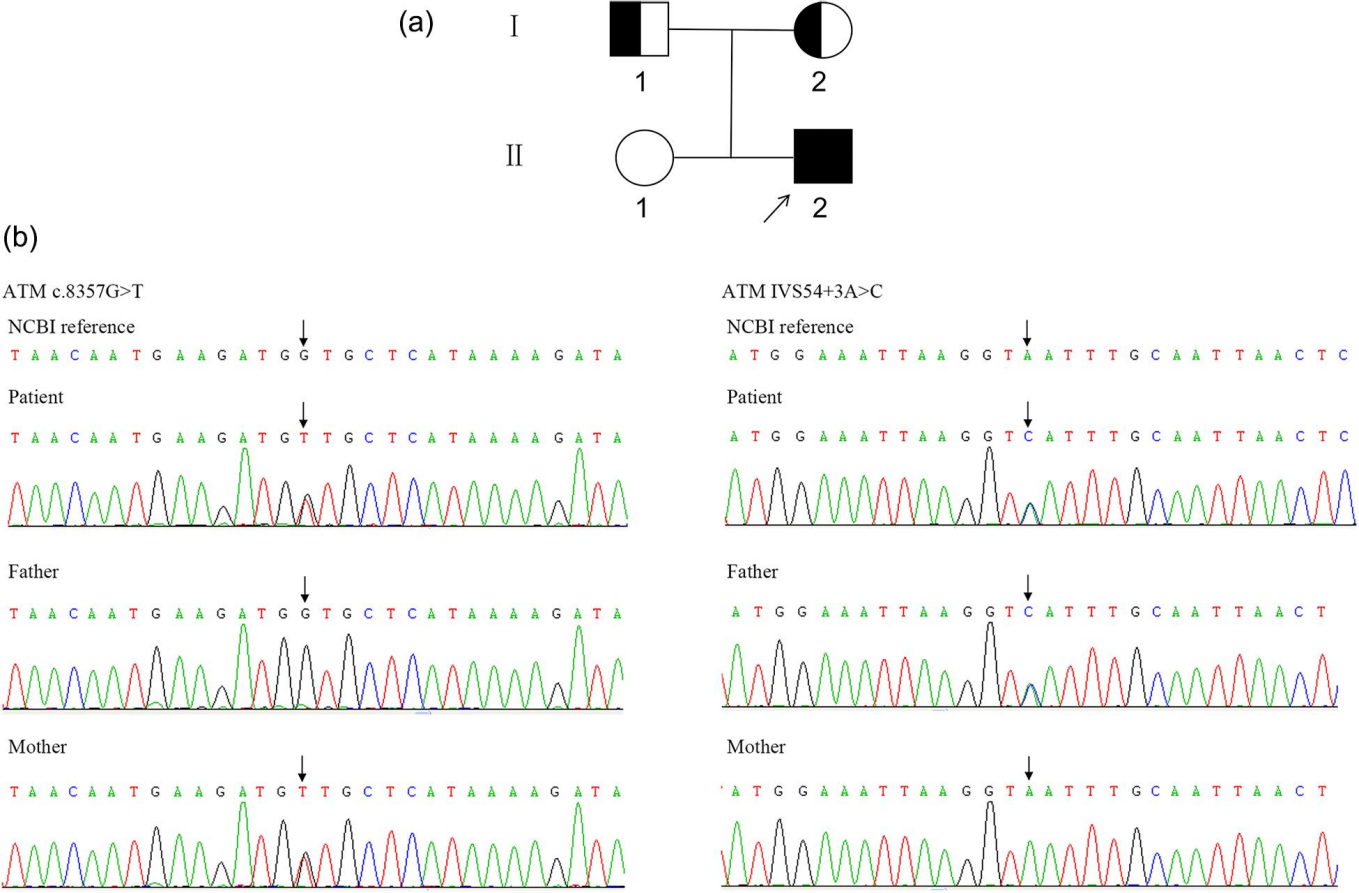
ATM mutations and identification of affected families. **(a)** The pedigree of the family: The arrow indicates the proband; his parents and elder sister have no signs of AT. **(b)** The variants detected in the family: The proband has both variants, while the c.8357G>T variant was only detected in his mother and the IVS54+3A>C variant was only detected in his father.

Genetic sequencing of the *ATM* gene identified novel compound heterozygous variants (Figure 1):

1. c.8357G>T (p.Gly2786Val): Inherited from the mother, predicted to be deleterious.
2. IVS54+3A>C: Inherited from the father, a splice site variant.

Both variants are novel to the best of our knowledge. Based on the clinical phenotype and sequencing results, a diagnosis of AT was established.

**Figure 2:**
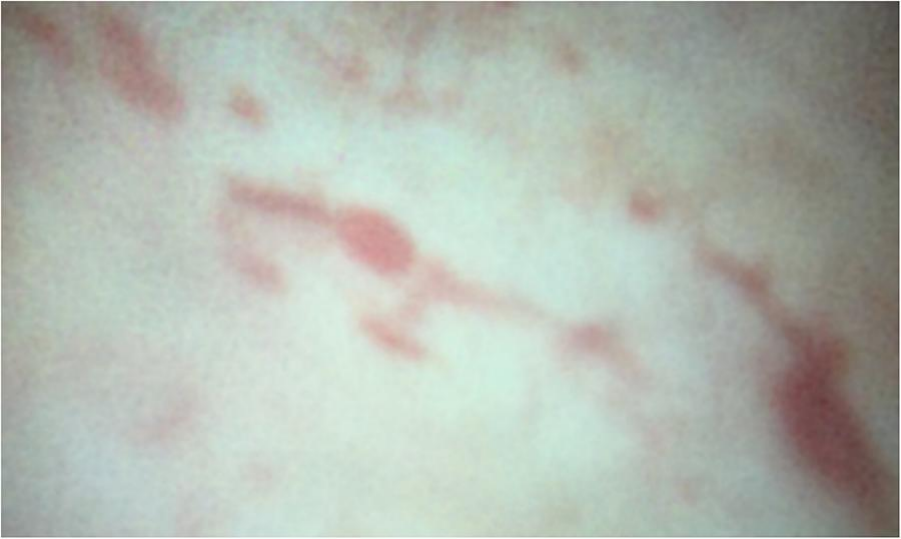
Cystoscopy image.

Based on the clinical manifestations and exclusion of other causes, a diagnosis of hemorrhagic cystitis was made. Cystoscopy revealed vascular dilation (telangiectasia) of the bladder wall with widespread yellow-brown sedimentations (Figure 2). The lesions bled easily upon contact, and no neoplastic changes were observed.

Approximately 1 month later, the patient was readmitted due to massive hematuria. Emergency surgery with electrocoagulation was performed.

### Outcome and follow-up

The hematuria resolved following the electrocoagulation procedure.

## Discussion

AT is typically classified as classic or variant based on age at onset, disease progression, and residual ATM activity. The diagnosis of classic AT rests on a constellation of features including early-onset progressive cerebellar ataxia, oculocutaneous telangiectasia, immunodeficiency, and cancer predisposition. Our patient had a typical history of early-onset ataxia, cerebellar atrophy on imaging, scleral telangiectasia, immunoglobulin deficiency, and a history of leukemia consistent with classic AT.

Most pathogenic ATM genotypes involve homozygous or compound heterozygous variants; nearly 1,000 different pathogenic variants have been cataloged. In classic AT, truncating (nonsense and frameshift) variants are common. We evaluated the variants according to the 2015 ACMG/AMP guidelines: Both variants are absent from control populations in the Exome Sequencing Project and 1000 Genomes Project, and are exceedingly rare in the gnomAD database (v4.0.0), with a combined allele frequency of <0.0001% (PM2). Multiple computational predictions support a deleterious effect (PP3). Although these variants are currently classified as variants of uncertain significance (VUS) due to lack of prior reports and functional data, the patient's clinical phenotype strongly supports their pathogenicity.

Defects in DNA repair and immunodeficiency contribute to increased cancer susceptibility in AT patients. Approximately one-third of AT patients develop malignancies, a risk about 25% higher than that of the general population (2). Children with AT are more susceptible to leukemia and lymphoma, while adults are prone to solid tumors. The prognosis of AT-associated tumors is often poor. This patient achieved complete remission after chemotherapy, though long-term surveillance is required.

Hematuria was the most prominent clinical feature in this case, characterized by (1) intermittent occurrence without an obvious trigger; (2) the presence of blood clots; (3) unremarkable upper urinary tract imaging; (4) isomorphic urinary red blood cells; and (5) normal coagulation and renal function. These findings strongly suggested a bladder origin. Cystoscopy confirmed multiple telangiectasias with active bleeding in the bladder wall, establishing the diagnosis of hemorrhagic cystitis.

Hemorrhagic cystitis may arise from several causes, most commonly viral infection (adenovirus, BK or JC polyomavirus) or exposure to alkylating agents such as CTX or IFO. Severe bladder hemorrhage is a rare complication of AT. Suzuki et al. reported the first case of vesical varices in an AT patient. To date, only 19 cases of hemorrhagic cystitis in AT patients have been reported across 10 articles (Table 1), most with prior CTX exposure. AT patients also have immunodeficiency, predisposing them to viral infections. For example, Christmann et al. reported a 14-year-old AT patient who developed severe hematuria 4 years after CTX treatment for B-cell lymphoma; cystoscopy showed bladder telangiectasia, and JC virus infection was confirmed (9). This was the first report of hemorrhage cystitis accompanied by JC virus infection in AT patients. However, viral-induced cystitis is typically self-limiting. Many experts attribute hemorrhagic cystitis in AT patients primarily to CTX toxicity—even at low doses—exacerbated by impaired DNA repair mechanisms. The latency between CTX exposure and hematuria can range from days to years (7). Furthermore, standard prophylaxis with hyperhydration and mesna appears insufficient to prevent this late-onset complication in AT patients (12). In our case, hematuria appeared 1.5 years after CTX treatment, suggesting that CTX may induce progressive telangiectatic changes in the bladder wall, leading to delayed, potentially massive hemorrhage.

**Table 1:** Etiologies and treatment of hemorrhagic cystitis in pediatric AT patients.

Case	Comorbidity	Duration after CTX	Pathogen in urine	Cystoscopy findings	Details of hematuria and treatment
1	ALL	8 h	None	Extensive hemorrhagic telangiectasia	Hematuria appeared 8 h after receiving CTX. No response to intravesical steroid, tranexamic acid, and intravesical cauterization. Resolved after selective arterial embolization (3).
2	BL	Not mentioned	BK virus	Hemorrhagic telangiectasia	Macroscopic hematuria. No response to bladder irrigation and tranexamic acid. Resolved by fulguration of the telangiectasias and evacuation of blood clots under cystoscopy (4).
3	BL	Years	None	Venous ectasia	Terminal and episodic macroscopic hematuria was noticed for a month. Hematuria improved spontaneously (5).
4	Not mentioned	Not mentioned	Not mentioned	Abundant telangiectasias	Intermittent macroscopic hematuria for 3 months. No response to monopolar coagulation and bladder washouts. Argon laser was used in open surgery on the bladder urothelium until a uniformly coagulated surface was achieved. The hematuria gradually decreased and disappeared by the fifth day after surgery (6).
5	ITP	Years	Not mentioned	Varicose veins in bladder	Massive hemorrhage in the bladder developed 3 years after completing CTX therapy. Treatment not mentioned (7).
6	ALL	Months	Not mentioned	Varicose veins in bladder	The patient developed massive hemorrhage. Treatment not mentioned (7).
7	HL	2 weeks	None	No need	Gross hematuria appeared 2 weeks after the second course of COPP (CTX dosage 600 mg/m^2^). The hematuria decreased and resolved spontaneously (8).
8	B-cell lymphoma	Years	JC virus	Severe hemorrhagic telangiectasia	The patient had been treated with corticosteroid for interstitial lung disease. Macrohematuria appeared. Cystectomy was applied for gross hematuria and rupture of the bladder (9).
9	BL	Years	Not mentioned	Prominent submucosal vessels	Intermittent hematuria requiring blood transfusion, cystoscopic removal of bladder clots occasionally. No response to irrigation of the bladder with normal saline or alum. Bleeding improved after a Bugby electrode was used to diathermy the vessels (10).
10	B-cell lymphoma	Years	Not mentioned	Multiple hemorrhagic telangiectasia, clots	Initially brief hematuria, got worse within 4 years and required repeat blood transfusions every 3–4 days. A large clot was removed from the bladder, and the multiple bleeding points were diathermied with a Bugby electrode which stopped the bleeding (10).
11	ITP	Years	Not mentioned	Mucosal inflammation with severe telangiectasias	Gross hematuria with bladder tamponade. No response to intensive treatments, repeated selective embolization of the vesical arteries and diathermocoagulation of the vassal lesions. Cystotomy was performed. Microhematuria and occasional severe gross hematuria can be observed even at the 10 months after the operation (11).
12	B-cell NHL	Months	None	Multiple telangiectasias, clots	Massive, painless hematuria requiring blood transfusion for two times. A large clot was removed followed by catheter irrigation of the bladder with regular saline solution, and bilateral ureteral stents were placed. Hematuria improved gradually (12).
13–19	leukemia or lymphoma	Not mentioned	Not mentioned	Not mentioned	Seven of 14 (50%) patients exposed to 1,200 mg/m^2^ or greater of CTX suffered hemorrhagic cystitis, with serious complications in three patients. Alternative explanations or etiologies for the cystitis were not found. All received intravenous hydration but none received mesna. Three patients had severe complications and received urologic intervention (13).

ALL, acute lymphoblastic leukemia; BL, Burkitt's lymphoma; ITP, idiopathic thrombocytopenic purpura; HL, Hodgkin lymphoma; NHL, Non-Hodgkin lymphoma.

The ATM gene plays critical roles in preventing cellular senescence and tumorigenesis. At the same time, this gene is essential for the formation of immature vessels in response to accumulation of reactive oxygen species (ROS), as well as for maintaining vascular stability, which is through oxidative activation of p38α rather than through the DNA repair pathway (14). As previously mentioned, most cases of severe hemorrhagic cystitis involve the use of CTX. It is well known that CTX causes hemorrhagic cystitis, primarily due to accumulation of its metabolite acrolein in the urine. Oxidative stress is one of the key mechanisms underlying the toxic effects of acrolein, which may contribute to vascular endothelium and blood vessel damage in the bladder wall. Although direct evidence is lacking, we speculate that defects in the ATM gene may limit the ability of affected individuals to repair the vascular damage caused by CTX, thereby laying the groundwork for recurrent bladder bleeding in the future.

Management of hemorrhagic cystitis depends on severity. Conservative measures and bladder irrigation with hemostatic agents may suffice for mild cases. Persistent or severe hemorrhage may be life-threatening and require surgical treatment, which may include cystoscopic electrocoagulation, laser therapy (e.g., Nd:YAG), transarterial embolization of the bladder artery, or, rarely, cystectomy (3). In this patient, cystoscopic electrocoagulation achieved hemostasis.

## Conclusion

The c.8357G>T and IVS54+3A>C variants in the *ATM* gene are pathogenic for AT. Patients with AT are at risk for hemorrhagic cystitis due to bladder wall telangiectasia, which can lead to life-threatening hemorrhage. This complication may be associated with prior chemotherapy but can present with significant latency. Cystoscopy is essential for diagnosis and enables early intervention. Surgical treatment, such as electrocoagulation, may be necessary for severe cases.

## Data Availability

The original contributions presented in the study are included in the article/Supplementary Material, further inquiries can be directed to the corresponding author.
